# Susceptibility to Postbiotic Substances-Enterocins of the Strains *Enterococcus thailandicus* from Beavers (*Castor fiber*)

**DOI:** 10.3390/pathogens14030269

**Published:** 2025-03-11

**Authors:** Andrea Lauková, Valentína Focková, Marián Maďar, Renata Miltko, Monika Pogány Simonová

**Affiliations:** 1 Centre of Biosciences of the Slovak Academy of Sciences, Institute of Animal Physiology, Šoltésovej 4-6, 040 01 Košice, Slovakia; valentina.fockova@gmail.com (V.F.); simonova@saske.sk (M.P.S.); 2 Department of Microbiology and Immunology, University of Veterinary Medicine and Pharmacy in Košice, Komenského 73, 041 81 Košice, Slovakia; marian.madar@uvlf.sk; 3 The Kielanowski Institute of Animal Physiology and Nutrition, Polish Academy of Sciences, Instytucka 3, 05 110 Jablonna, Poland; r.miltko@ifzz.pl

**Keywords:** beaver, postbiotic, biofilm, susceptibility, *Enterococcus*

## Abstract

Eurasian beaver (*Castor fiber*) populations have been reintroduced to European countries, though this bears the risk of novel wildlife pathogen reservoir establishment. The species nova *E. thailandicus* was described first in Thailand as a food-derived strain. Later, this species was detected in the feces of pigs, poultry, sewage, and humans. In those studies, the potential risk posed by this species was evaluated. Against that background, the aim of this study was to investigate the susceptibility to postbiotic active substances (enterocins) against fecal *E. thailandicus* strains from beavers caught in Poland. The strains were identified with the use of 16S rRNA gene similarity sequencing. These six *E. thailandicus* strains with low-grade biofilm-forming abilities and two strains with the presence of the *gelE* virulence factor gene were susceptible to seven enterocins produced by non-autochthonous strains, mostly of the species *E. faecium* and *E. durans*. The inhibitory activity against the tested strains reached 25,600 AU/mL. Although the studied *E. thailandicus* strains did not show a strictly pathogenic character, their growth inhibition by postbiotics was identified as a novel elimination strategy.

## 1. Introduction

Monitoring Eurasian beaver (*Castor fiber*) populations is key because their reintroduction to European countries bears risks associated with their importation, including the emergence of wildlife diseases and zoonoses, which can result in the establishment of novel wildlife pathogen reservoirs [1,2]. In Poland, the future status of the European beaver is unclear, as the beaver population’s uncontrolled increase is costly and raises conflicts when beavers’ activities infringe upon the intended use of land by humans [3]. However, since the beginning of the 21st century, efforts have been made to manage the Polish beaver population by hunting [3]. In that context, in our study, *E. thailandicus* strains were isolated from samples of beavers captured in Poland [4,5,6]. The strain FP48-3^T^ (isolated from fermented sausage) was first described in Thailand as a novel taxon *E. thailandicus* [7]. Its allotment to the novel species *E. thailandicus* was confirmed by assessing its complete 16S rRNA gene sequence [7]. The researchers noted the potential clinical significance of this species. Enterococci generally have the dual characteristics of being opportunistic pathogens and beneficial strains [8]. For example, Wu et al. [8] detected the *E*. *thailandicus* strain TC1, isolated from healthy pigs, and reported its possible association with health risks. Wigmore et al. [9] revealed a high incidence of *E. thailandicus* when testing enterococci from poultry in Victoria (Australia). In addition, Ybazeta et al. [10] described the complete genome sequence of *E. thailandicus* strain a523 isolated from raw urban sewage. Moreover, Mbouche et al. [11] reported an *E. thailandicus* species strain as an unusual pathogen encountered in humans with intra-abdominal infection. Based on our previous characterization studies regarding the virulence factor genes (*adhesin*s) in *E. thailandicus* [5,6], in this study, beaver-derived *E. thailandicus* strains were analyzed to determine their susceptibility to postbiotics.

Postbiotics are a complex preparation containing many bioactive compounds with multiple mechanisms of action [12,13,14]. Two major mechanisms by which postbiotics can offer a clinical benefit are immune system modulation and enhancement of the intestinal barrier function [12,13]. A hypocholesterolemic effect was found in broiler rabbits and the tendency to improve phagocytic activity and jejunal morphometric parameters [14]. Stimulation of phagocytosis was also found in the case of experimental trichinellosis in mice, which can contribute to decreased larval migration and a reduced parasite burden in the host [15]. The extant research has noted that in vitro and in situ effects should be mapped, to reduce the influence of non-target microbial agents. The research on postbiotics is intensifying, with the goal of facilitating their introduction to prevent and/or treat disease [16]. Al-Madboly et al. [17] presented bacteriocin– enterocin LNS18, an active anti-cancer (against HepG2 cell line) postbiotic produced by strain of the *E. thailandicus* species. To extend such findings, the primary aim of this study was to find possible ways to eliminate/reduce *E. thailandicus* strains’ potential risks (e.g., virulence factor genes, biofilm formation) using postbiotics by first identifying strains’ susceptibility to these postbiotics. This work fits within the broader scientific context of studying inhibitory spectra of postbiotics against species and strains from different sources. These studies will help manage risks in the future and have implications in that regard for the beaver ecosystem.

## 2. Materials and Methods

### 2.1. Sample Treatment

Our colleagues caught free-living beavers in northeast Poland [5] (12, male and female, aged 4–5 years) according to the ethical rules for animal handling in the Województwo (Province) Podlaskie, Gmina Wizajny, GPS: 22 52 E: 54 22 N. They were caught with a net and placed in wire cages. The sex of each beaver was determined based on the secretion of their anal glands (Cool Gray 1U-2U and 609U-610U-Pantone matching system, NJ, 1991). The average body weight of the beavers was 18. 8 ± 2.2 kg and the average length 111.2 ± 4.2 cm. The beavers were euthanized by an injection of 2.5 mL xylazine and 1 mL ketamine (Biowet, Poland). The administered doses were consistent with the procedure for farm-grown beavers adopted by the Research Station in Popielno (Polish Academy of Sciences). The feces (12), colon (12), and caecum (6) were sampled, transported to our laboratory, and treated with the standard microbiological method (ISO, International Organization for Standardization). Selected, isolated colonies were identified using matrix-assisted laser desorption/ionization time-of-flight mass spectrometry (MALDI-TOF MS, Bruker Daltonics, San Jose, CA, USA), as previously reported by Lauková et al. [5]. The taxonomic allotment of the six identified strains of *E. thailandicus* showed evaluation scores in a range from 1.869 to 2.337 [5]. Next, the strains were submitted to BLASTn (basic local search tool) for homology analysis of the 16S rRNA gene sequence (http://blast.ncbi.nlm.nih.gov/blast.cgi, accessed on 22 May 2022). 

### 2.2. Species Taxonomic Identification Using Sequence Analysis

The genus and species allocation of the strains was determined using BLASTn (basic local search tool) homology analysis (http://blast.ncbi.nlm.nih.gov/blast.cgi, accessed on 22 May 2022) of the 16S rRNA gene sequence, which was amplified using the universal primers Bac27F (5′-AGAGTTTGATCCTGGCTCAG-3′) and 1492R (5′-CGGTTACCTTGTTACGACTT-3′, Merck-Sigma Aldrich, Darmstadt, Germany) as described in detail previously by Focková et al. [18]. DNAzol direct (Molecular Research Centre Inc., Cincinnati, OH, USA) was used to extract the genomic DNA from a pure solitary colony. Amplified products were sent for purification in a low bind tube at a minimal volume of 15 µL and for sequencing in both directions using the formerly indicated primers. The PCR mixture (50 µL) per sample contained 2 µL of DNA shield, 46 µL of a reaction mixture comprising One Taq 2x Master Mix with Standard Buffer (New England Biolabs, United Kingdom), diluted with water for molecular biology (PanReac AppliChem, Darmstadt, Germany) to 1 × concentration, and 1 µL of each primer (33 µM). The reactions were performed on a TProfesional Basic thermocycler (Biometra GmbH, Goettingen, Germany) according to the following protocol: 5 min at 94 °C, followed by 30 cycles at 94 °C for 1 min, annealing at 55 °C for 1 min, followed at 72 °C for 3 min, and a final extension step at 72 °C for 10 min. Aliquot PCR products were analyzed with 3% (*w*/*v*) agarose gel electrophoresis in Tris-acetate-EDTA buffer (pH 7.8). They were visualized with GelRed (Biotium Inc., Hayward, CA, USA). The 16S rRNA sequences were validated and assembled using Geneious 8.05 (Biomatters, Auckland, New Zealand) and subjected to BLASTn analysis as indicated. This method uses the highly conserved nature of the 16S ribosomal RNA (rRNA) gene in all prokaryotes, containing variable regions that differentiate between species.

### 2.3. Strains’ Growth on Different Agar Media

The growth of strains on different media also contributed to their species identification. *E. thailandicus* strains’ growth on the following media was tested: Kanamycin–esculin-azide agar (Oxoid, UK), KF–Streptococcus agar (Becton and Dickinson, Cockeysville, MD, USA), M–Enterococcus agar (Difco, Atlanta, GA, USA), de Man–Rogosa–Sharpe (MRS) agar (Merck, Darmstadt, Germany), brain–heart agar (BHA, Difco, Atlanta, GA, USA), and Mueller–Hinton agar (Becton Dickinson). Each agar was inoculated with the tested strain, and the growth colonies were checked in two replicates. The character and intensity of a grown strain were assessed as either excellent, sufficient, or no growing strain. As a positive control, the strain DSM21767TJUG was used.

### 2.4. Biofilm-Forming Ability of E. thailandicus Strains

The quantitative microtiter plate assay was used according to Chaieb et al. [19], as previously described by Focková et al. [20]. A pure colony of the tested strain grown on Brain–heart agar overnight (Difco, Sparks, MD, USA) was transferred to 5 mL of Ringer solution (pH 7, Merck, Darmstadt, Germany) to the amount of 1.0 × 10^8^ CFU/mL. An aliquot of 100 µL from that suspension was transferred into 10 mL of Brain–heart infusion (BHI). A 200 µL aliquot of the dilution was inoculated in microtiter plate wells (Greiner ELISA 12 Well Strips, 350 µL flat bottom, Frickenhausen GmbH, Frickenhausen, Germany). The plates were incubated at 37 °C for 24 h. The biofilm forming in the microtiter plate wells was washed twice with 200 µL of deionized water and dried at 25 °C for 30 min. The remaining bacteria were stained for half an hour at 25 °C with 200 µL of 0.1% (*w*/*v*) crystal violet in deionized water. Then, the wells were washed twice with a 200 µL volume of deionized water. The plate was again dried (30 min at 25 °C). The dye bound to the adhered biofilm was extracted with 200 µL of 95% ethanol and stirred. A 150 µL aliquot was transferred to a new microplate well to measure absorbance (A) at 570 nm (Synergy TM4 MALDI Mode Microplate reader, Biotek, Seattle, WA, USA). Each strain and condition were analyzed in two independent analyses with 12 replicates. A sterile BHI broth was included in each test as a negative control. *Streptococcus equi* subsp. *zooepidemicus* CCM7316 was used as a positive control (provided by Dr. Styková from the University of Veterinary Medicine and Pharmacy in Košice, Slovakia). The biofilm-forming ability was evaluated as highly positive (A_570_ ≥ 1), low-grade positive (0.1 ≤ A_570_ < 1), or negative (A_570_ < 0.1), as reported by Chaieb et al. [19].

### 2.5. Detection of Virulence Factor Genes—Gelatinase (gelE), Esp, and Agg

Based on our previous results [6], the presence of the following virulence factor genes was analyzed according to Kubašová et al. [21]: *gelE* (gelatinase), *esp* (enterococcal surface protein), and *agg* (aggregation substance). The DNA was extracted using rapid alkaline lysis [22]. Briefly, DNA was prepared from the purified culture. A 1 µL loopful of cells was suspended in 20 µL of lysis buffer (0.25%SDS, 0.05 N NaOH) and heated at 95 °C for 5 min. The cell lysate was spun down by brief centrifugation at 16,000× *g* and diluted by adding 180 µL of distilled water. The cell debris was removed by centrifugation at 16,000× *g* for 5 min. Supernatants were directly used as the template for PCR or were frozen at −20 °C until further use. We used 25 µL for PCR, where the mix consisted of 1 × reaction buffer, 0.2 mmol/L of deoxynucleoside triphosphate, 3 mmol/L MgCl_2_, 1 µmol/L of each primer, 1U of Taq DNA polymerase, and 1.5 µL of DNA template. The cycling conditions included an initial step of 95 °C for 3 min, 35 cycles of 30 s at 95 °C, 30 s at 55 °C, 30 s at 72 °C, and a final step at 72 °C for 5 min. The PCR products were separated by agarose gel electrophoresis (1.2% *w*/*v*, Sigma-Aldrich, Saint Louis, MO, USA) containing 1 µL/mL ethidium bromide (Sigma-Aldrich) in 0.5 × TAE buffer (Merck, Darmstadt, Germany). The PCR fragments were visualized with UV light. The positive control strains were *E. faecalis* P36 and *E. faecium* F10 (kindly provided by Dr. Teresa Semedo-Lemsaddek, University of Lisbon, Portugal).

### 2.6. Susceptibility of E. thailandicus Strains to Enterocins (Postbiotic Substances)

The postbiotic substances (precipitates, partially purified substances) produced by the strains isolated and characterized in our laboratory were used to test susceptibility: Enterocin (Ent) A/P produced by the environmental strain EK13 = CCM7419 [23] is a heat-stable dipeptide with a molecular mass of 4830 Da. Its optimum production is in the pH range of 5.0–6.5 at 30–37 °C. The most active substance is produced in the log growth phase of the producer strain. It has a broad inhibitory spectrum. Ent M is also produced by the environmental strain *E. faecium* AL41 = CCM8558 [24]. It is a thermo-stable, small peptide with molecular mass of 4628 Da, possessing a broad antimicrobial spectrum. Ent 412, produced by the horse-derived strain *E. faecium* EF412 [25], represents a broad antimicrobial substance. Ent 4231, produced by the ruminal strain *E. faecium* CCM 4231 [26], is the first characterized enterocin of an animal/ruminant-derived producing strain with a broad antimicrobial spectrum, sensitive to pronase and resistant to heating. Ent 55 is produced by the poultry (crop of chicken) strain *E. faecium* EF55 [27]. It is thermo-stable bacteriocin with the highest production in the late logarithmic growth phase, optimum production in the pH range of 7.0–9.0, and inhibitory activity mostly against Gram-positive bacteria. Ent 2019 is produced by the rabbit-derived strain *E. faecium* 2019 (CCM 7420) [28], and Durancin ED26E/7 is produced by the food-derived strain *E. durans* ED26E/7 [29]. The Ents used belong to class II of the enterocins [30]. They were prepared according to formerly reported protocols. The testing method was the agar spot method, according to De Vuyst et al. [31]. Briefly, BHagar (Difco) was used as the basic agar. An overnight broth culture of the EA5 strain (A_600_ up to 1.0) (200 µL) was applied to 0.7% BHagar and the plate was overlaid. The appropriate dilutions of enterocins (in phosphate buffer, pH 6.5, 10 µL) were dropped on the agar plate surface. The plates were stored for 10 min in the fridge to diffuse the enterocin. Then, the plates were incubated at 37 °C overnight, first checking them after 4 h. The inhibitory activity was expressed in arbitrary units per mL (AU/mL), meaning a two-fold dilution (in phosphate buffer pH 6.5) of precipitate, which inhibited the growth of the indicator strain. The initial activity of the precipitate was measured against the principal indicator *Enterococcus avium* EA5 (our strain; Ent M, Ent 2019–25,600 AU/mL, Ent A/P, Ent 55–51,200 AU/mL, Ent 412–204,800 AU/mL, ED26E/7 Ent 4231–6400 AU/mL).

## 3. Results

### 3.1. Sequence Analysis Taxonomic Allotment

BLASTn 16S rRNA sequence analysis confirmed that the taxonomy of the isolated strains (previously identified by MALDI-TOF MS spectrometry) belonged to the species *E. thailandicus* (Table 1), with the sequence gene similarity/identity from 99.60% to 100%. Sequence gene similarity (NR_114015.1) was found with *E. thailandicus* NBRC 101067 in GenBank. The % abundance among strains was well-balanced; the strain ETr10/2 was found to have the highest similarity (100.0%), although its score value revealed by MALDI-TOF MS spectrometry indicated only probable genus identification. In the opposite case, the strain ET10/1 with the highest MALDI-TOF MS score (2.337), indicating highly probable species identification, showed 99.60% abundance, similar to the strains ET10/2 and ETr10/1 (Table 1). A higher % abundance was also noted in the strains ET12/2 (99.72%) and ET12/1 (99.81%).

### 3.2. Detection of Virulence Factor Genes Among E. thailandicus Strains and Their Grown on Different Media

The growth of all tested *E. thailandicus* strains on MRS agar, MH agar, and Kanamycin–esculin–azide agar was sufficient. When using KF Streptococcus agar as well as M–Enterococcus agar, the strains’ growth was excellent. The colonies on KF–Streptococcus agar showed significant growth as dark red/burgundy colonies. Growth of *E. thailandicus* strains on M–Enterococcus agar, meanwhile, was characterized by dark purple/burgundy and round/ovoid colonies. When using Kanamycin–esculin–azide agar, colonies were small and white–gray with black zones. The growth on BHagar was also excellent, with typical white colonies.

Among *E. thailandicus* strains, the *agg* and *esp* genes were absent; the *gelE* gene was present in the strains ET12/1 and ET12/2 (Table 2), while the other strains were *gelE* gene-free.

The strains of *E. thailandicus* were found to have low-grade biofilm-forming ability, which ranged from 0.126 ± 0.36 to 0. 415 ± 0.65 (Table 2). This ability was well-balanced, with the lowest value measured in the strain ET12/2 (0.126 ± 0.36) and the highest in the strain ETr10/1 (0.415 ± 0.65). Finally, the strains were submitted testing for their susceptibility to postbiotic substances, that is, bacteriocins (enterocins).

### 3.3. E. thailandicus Strains’ Susceptibility to Enterocins (Postbiotic Substances)

Biofilm-forming *E. thailandicus* strains (including two strains possessing the *gelE* gene) were found to be susceptible to seven enterocins, with almost the same inhibitory activity up to 25,600 AU/mL (Table 3). As exceptions, Ent 4231 reached inhibitory activity at 12,800 AU/mL and Durancin ED26E/7 reached inhibitory activity at 6400 AU/mL.

## 4. Discussion

The strains taxonomy can be identified through different methods. The 16S rRNA gene sequence is the most commonly used genetic marker for studying bacterial taxonomy and phylogeny. The principle of 16S rRNA gene sequencing is based on amplicon sequencing, which targets specific regions of the genome. The genetic information in the 16S rRNA gene after sequencing is used for identification [32]. In our case, it was important to verify that formerly performed MALDI-TOF MS identification (although with different score values) was in accordance with next-generation sequencing identification. This technique (16S rRNA) was also used to identify the *E. thailandicus* TC1 strain from healthy pigs [8]. The TC1 strain was revealed by β-hemolysis similarly, as previously described for our *E. thailandicus* strains by Cibulková [33]. TC1 was found to have susceptibility to antibiotics. Regarding identification, other species’ next-generation sequencing results were also in accordance with their MALDI-TOF MS identification, e.g., *E. mundtii* from the horse breed Norik from Muráň [20]. *E. thailandicus* species have rarely been isolated from fecal and/or clinical samples [7,8]. In our previous study, beavers were found to be a source of this species [5], and detection was also performed in healthy pigs [8]. Other strains of the species *E*. *thailandicus* were food-derived, isolated from milk and/or fermented sausage [7,8].

The growth possibility on different media is a parameter that can be associated with the characteristics of a strain and/or (as in the case of, e.g., bacteriocin-producing strains) regarded as important as a condition for its purification process. A suitable purification process can depend on selecting media where bacteria can produce the most bacteriocin substance. Tanasupawat et al. [7] described the first strain of the species nova *E. thailandicus* with sufficient growth on Kanamycin–esculin–azide agar. In the present study, *E. thailandicus* was studied from another perspective. Elsewhere, Cibulková [33] found *E. thailandicus* strains with low potential for bacteriocin activity. However, given the susceptibility to enterocins of the *E. thailandicus* strains tested here, we found that they were inhibited with enterocins produced by non-autochthonous strains, mostly *E. faecium* but also *E. durans*. Bacteriocins (previously indicated to belong to postbiotics) are peptides with a cationic nature [17]. Also the Enterocin LNS18 was reported produced by the strain *E. thailandicus* isolated from a fermented dairy product in Egypt. Furthermore, Ent A/P, Ent M, Ent 2019, and Ent 55 were even found to be active against Gram-negative bacteria from horses. They even inhibited bacterial species such as *Pantoea agglomerans*, *Sfingomonas paucimobilis*, *Aeromonas caviae,* and *Yersinia* spp. [34]. Enterocins used in this study were also active against methicillin-resistant staphylococci from rabbits, dogs, fishes, horses and even beavers, with inhibitory activity from 100 to 25,600 AU/mL [35]. Bacteriocins (postbiotic substances) can act by different mechanisms. Enterocins are usually allotted to class II, to which the substances tested here belong. Their antimicrobial destruction potential arises through interaction with and disturbance of the bacterial cytoplasmic membrane [36]. The advantages of bacteriocins are that they are effective at lower concentrations and that their degradation products are easily metabolized with no side effects [37]. Recently, an antibiofilm effect of bacteriocins as a postbiotic was also reported [38]. In general, it can be concluded that postbiotics are safe and stable with a long shelf-life, enabling easy storage and transportation with a real reducing effect. Accordingly, we can recommend them as a novel reducing/eliminating approach for antimicrobial treatment [12]. In this context, it is recommended, other *E. thailandicus* must be tested for their susceptibility to enterocins or other bacteriocins. Despite that, based on each case reported, this study indicates we have uncovered a promising approach.

## 5. Conclusions

This study identified susceptibility to seven postbiotic substances, enterocins (produced by non-autochthonous strains *E. faecium* and *E. durans*), of six identified *E. thailandicus* strains from beavers possessing low-grade biofilm-forming ability, with two strains (ET12/1, ET12/2) having the virulence factor *gelE* gene. Inhibitory activity reached 25,600 AU/mL. Although the tested *E. thailandicus* did not show strictly pathogenic potential, the use of effective enterocins to eliminate those strains has been identified as having potential for use in the future as a novel approach. The novelty of this study lies in our use of original enterocins produced by the strains and their substances isolated, characterized and studied in our laboratory.

## Figures and Tables

**Table 1:** Evaluation scores and percentage abundances of identified *E. thailandicus* strains.

*E. thailandicus*	MALDI-TOF MS Score	% Abundance (BLASTn Analysis)
ET10/1	2.337	99.60% (NR_114015.1)
ET10/2	1.862	99.61% (NR_114015.1)
ET12/1	2.137	99.82% (NR_114015.1)
ET12/2	2.143	99.72% (NR_114015.1)
ETr10/1	1.981	99.60% (NR_114015.1)
ETr10/2	1.869	100.0% (NR_114015.1)

Evaluation scores from MALDI-TOF MS, as previously presented by Lauková et al. [5]. The control strain was DSM21767TJUG (Bruker Daltonic, 2008). Range description: 1700–1999, probable genus identification; 2000–2299, secure genus identification/probable species identification; 2300–3000, highly probable species identification; percentage (%) abundance gene similarity for the sequence of type/reference strain *E. thailandicus* NBRC 101067.

**Table 2:** Biofilm-forming ability and *gelE* gene occurrence in *E. thailandicus* from beavers.

*E. thailandicus*	*gelE* Gene	Biofilm (±SD)
ET10/1	-	0.131 ± 0.36
ET10/2	-	0.137 ± 0.37
ET12/1	+	0.162 ± 0.40
ET12/2	+	0.126 ± 0.36
ETr10/1	-	0.415 ± 0.65
ETr10/2	-	0.257 ± 0.50

Biofilm: highly positive (A_570_ ≥ 1), low-grade positive (0.1 ≤ A_570_ < 1), or negative (A_570_ < 0.1); *gelE* gene—gelatinase; in the strains, *esp* and *agg* genes were absent. Positive control strain *Str. equi* subsp. *zooepidemicus* had biofilm-forming ability with a value of 0.128 ± 0.36.

**Table 3:** Susceptibility to enterocins (postbiotic substances) of *E. thailandicus* strains from beavers (inhibitory activity is expressed in arbitrary units per mL, AU/mL).

*E. thailandicus*	Ent A/P	Ent M	Ent 412	Ent 4231	Ent 55	Ent 2019	DurED26E/7
ET10/1	25,600	25,600	25,600	12,800	25,600	25,600	6400
ET10/2	25,600	25,600	25,600	12,800	25,600	25,600	6400
ET12/1	25,600	25,600	25,600	12,800	25,600	25,600	6400
ET12/2	25,600	25,600	25,600	12,800	25,600	25,600	6400
ETr10/1	25,600	25,600	25,600	12,800	25,600	25,600	6400
ETr10/2	25,600	25,600	25,600	12,800	25,600	25,600	6400

Ent A/P, enterocin produced by the environmental strain EK13 = CCM7419 [23]; Ent M, produced by the strain *E. faecium* AL41 = CCM8558 [24]; Ent 412, produced by the horse-derived strain *E. faecium* EF412 [25]; Ent 4231, produced by the ruminal strain *E. faecium* CCM4231 [26]; Ent 55, produced by the poultry strain *E. faecium* EF55 [27]; Ent 2019, produced by the rabbit-derived strain *E. faecium* 2019 (CCM 7420) [28]; Dur ED26E/7, produced by the food-derived strain *E. durans* ED26E/7 [29]; Ent M, Ent 2019–25,600 AU/mL, EntA/P, Ent 55–51,200 AU/mL, Ent 412–204,800 AU/mL, ED26E/7, Ent 4231–6400 AU/mL.

## Data Availability

Data can be provided by the corresponding author.
